# FITC-linked Fibrin-Binding Peptide and real-time live confocal microscopy as a novel tool to visualize fibrin(ogen) in coagulation

**Published:** 2017-05-24

**Authors:** Nikolaj Weiss, Bettina Schenk, Mirjam Bachler, Cristina Solomon, Dietmar Fries, Martin Hermann

**Affiliations:** 1 Department of Anesthesiology and Critical Care Medicine, Medical University Innsbruck, Innsbruck, Austria; 2 Department of General and Surgical Critical Care Medicine, Medical University Innsbruck, Innsbruck, Austria; 3 Institute for Sports Medicine, Alpine Medicine and Health Tourism, UMIT - University for Health Sciences, Medical Informatics and Technology, Hall, Austria.; 4 Department of Anesthesiology, Perioperative Medicine and General Intensive Care, Paracelsus Medical University, Salzburg, Austria; 5 Ludwig Boltzmann Institute for Experimental and Clinical Traumatology and AUVA Research Centre, Vienna, Austria

**Keywords:** coagulants, fibrin, fibrinogen, hemostasis, microscopy, confocal

## Abstract

**Background and Aim**: Although fibrinogen has been established as a key player in the process of coagulation, many questions about the role of fibrinogen under specific conditions remain. Confocal microscopic assessment of clot formation, and in particular the role that fibrinogen plays in this process, is commonly investigated using pre-labeled fibrinogen. This has a number of drawbacks, primarily that it is impossible to stain fibrin networks after their formation. The aim of the present study is to present an alternative for conveniently post-staining fibrin in a wide range of models/situations, in real time and with high resolution.

**Methods**: We combined a peptide known to bind fibrin and linked it to fluorescein isothiocyanate (FITC), creating the FITC-Fibrin-Binding Peptide (FFBP). We subsequently tested its fibrin-binding capability *in vitro* under static conditions, as well as under simulated flow, using real-time live confocal microscopy.

**Results**: Fibrin stained with FFBP overlaps with fibrin stained with fibrinogen pre-labeled with Alexa Fluor 647 following coagulation induction. In contrast to pre-labeled fibrinogen, FFBP also stains already formed fibrin networks. By combining FFBP with real-time live confocal microscopy even the visualization of single fibrin fibers is possible.

**Conclusions**: These data indicate that FFBP is a valid and valuable tool for real-time live confocal assessment of clot formation.

**Relevance for patients:** Our findings present a valuable alternative for the visualization of fibrin even after its formation, and we believe this approach will be particularly valuable for future investigations of important, but previously overlooked, aspects of clot formation.

## 1 Introduction

Understanding of clot formation and hemostasis has in-creased tremendously over the past decades as the specific molecular interactions between platelets, fibrin and other factors have been described in detail using methods such as con-focal microscopy in models ranging from *in vitro* models to *in vivo* intravital microscopy in rodents [1,2]. Although the unequivocal importance of fibrinogen in clot formation and hemostasis is something that has come to be universally recognized [3,4], many specific questions remain unanswered. The importance of developing tools for studying fibrinogen and fibrin formation was recently highlighted [5].

Fluorescently pre-labeled fibrinogen is a widely used option for confocal visualization of fibrin formation as it is incorporated into growing fibrin fibers [6-8]. The disadvantage of this approach is the necessity to add the pre-labeled fibrinogen while the network is being formed. Consequently, an existing fibrin network cannot be visualized using such an approach. This approach is also suboptimal for use in *in vivo* studies as the addition of the fluorescently labeled fibrinogen might influence fibrin formation, especially in cases where the levels of fibrinogen are low.

Here, we describe the usefulness of a fluorophore-conjugated fibrin-binding peptide in combination with real-time live confocal microscopy as a universal live fibrin visualization tool under static as well as simulated flow conditions. For this purpose, we linked a previously described fibrin-binding pep-tide with isothiocyanate and used real-time live confocal imaging for the visualization of fibrin networks [9,10]. The primary objective of this investigation was to validate the ability of such an approach to visualize in real time, in a confocal fashion, the formation of fibrin networks. The secondary objective was to test whether this approach is also well suited for live confocal imaging of fibrin networks after their formation. The promising preliminary results described here pave the way for the future use of this approach in coagulation analysis *in vitro* as well as *in vivo*, thereby helping to uncover important and previously overlooked aspects of clot formation and hemostasis [5].

## 2 Methods

### 2.1 FITC-Fibrin-Binding Peptide synthesis

Based on publications describing fibrin-binding peptides [9,10], we chose a previously described 11 amino acid long fibrin-binding peptide [10] and combined it at the amino terminus to fluorescein isothiocyanate (FITC). The amino acid sequence of the resulting peptide is (FITC)-tyrosine, D-glutamate, cysteine, hydroxyproline, L-3-chlorotyrosin, glycine, leucine, cysteine, tyrosine, isoleucine, glutamine-NH2 (TFA salt, disulfide bond Cys-Cys). We named it FITC-linked Fibrin-Binding Peptide (FFBP). The synthesis of the peptide and the linking to FITC was performed by GenicBio (Shanghai, China).

### 2.2 Overview of experiments performed with FFBP

**Figure 1:**
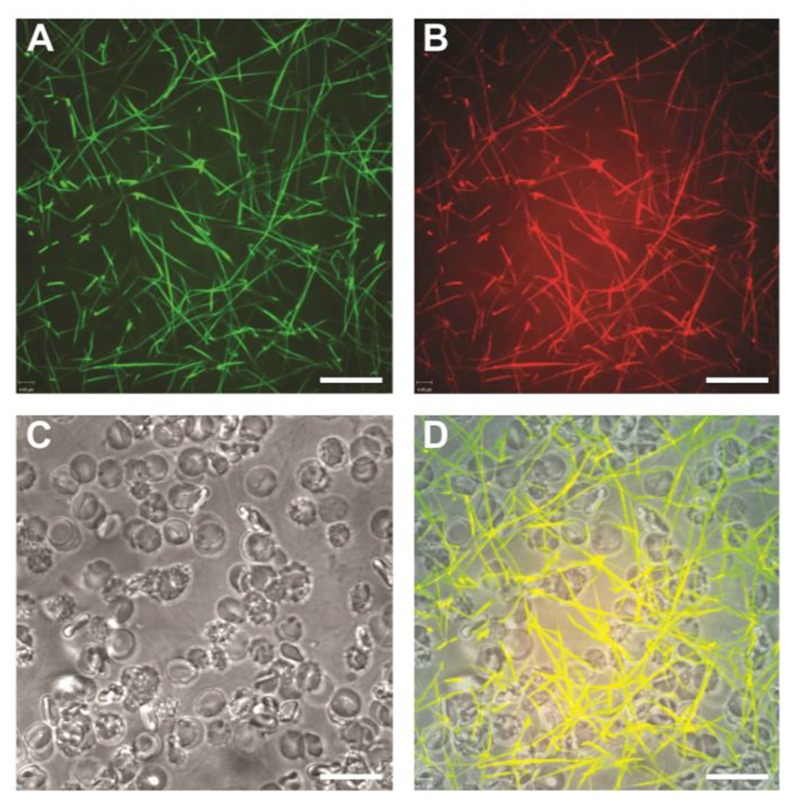
Comparison between FFBP and pre-labeled fibrinogen (Alexa Fluor 647). Clots were created from citrated human plasma with added RBCs and platelets by addition of star-tem/ex-tem. Shown are confocal images of fibrin fibers 1 µm above the bottom surface of the chamber stained with A) FFBP or B) pre-labeled fibrinogen; both images come from one sample stained with both methods. C) Shows a bright field image of RBCs/platelets and D) shows the merged image. Note the 100% overlay of the fibrin visualization with FFBP and pre-labeled fibrinogen resulting in the yellow color. Z-stack images (20 optical planes with a spacing of 0.2 µm) were acquired using a 63× oil immersion objective with a numerical aperture of 1.42. Scale bar = 16 µm. All experiments were performed at least three times, and representative samples are shown.

We performed the following specific assessments of FFBP:

1 – Fibrin binding under static conditions and comparison to pre-labeled human fibrinogen Alexa Fluor 647 (Thermo Fisher Scientific, Waltham, MA, USA) (Figure 1).

**Figure 2:**
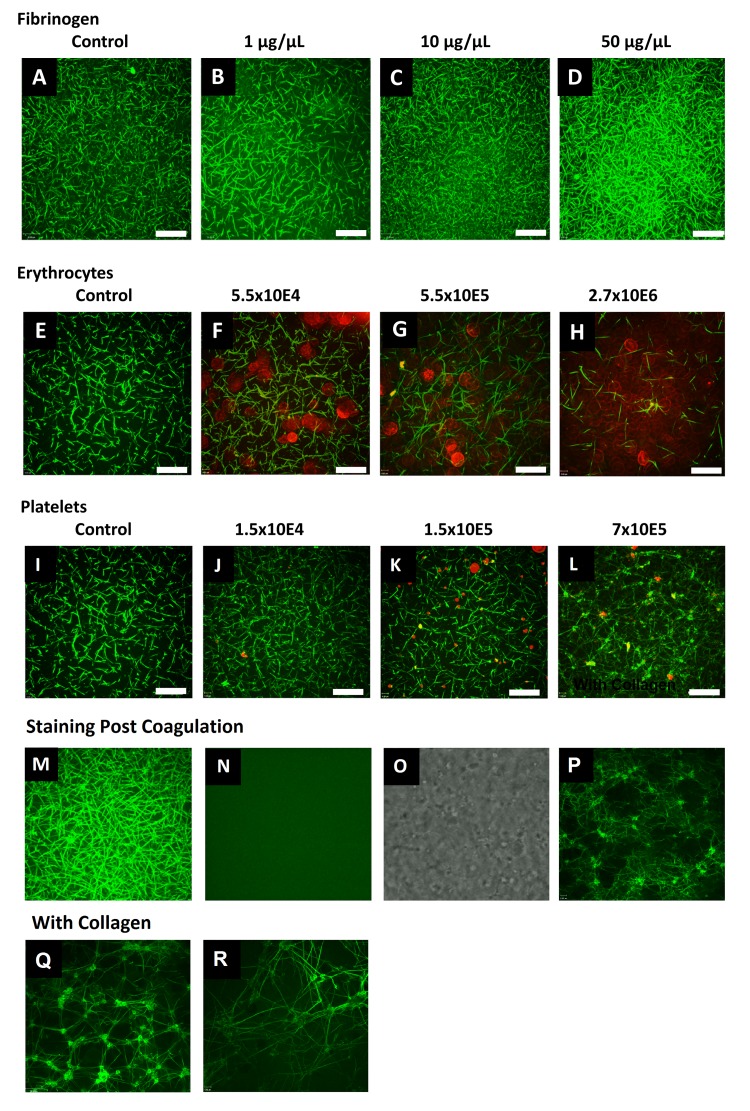
Clots from citrated human plasma were formed by addition of star-tem and ex-tem. All stainings were performed before induction of coagulation. The fibrin network is labeled in green via FFBP. Shown is the FFBP labeled fibrin network without (A) and with addition of fibrinogen (1/10/50 µg/µl; B-D). The influence of RBCs (5.5×10^4^-2.7×10^6^) on the fibrin network is shown in (E-H) and platelets (1.5×10^4^-7×10^5^) in (I-L). Fluorescence images of fibrin fibers were acquired 1 μm above the bottom surface of the chambers. In red the RBCs and platelets are visualized via wheat germ agglutinin (561 nm) staining. Merged images are shown. In panels M-O, coagulation was performed before the addition of the stains. Stains were added 15 min after starting the coagulation. M) Shows the result using FFBP; N) using pre-labeled human fibrinogen Alexa Fluor 647 and O) shows the same area as a bright field image in order to verify the presence of fibrin fibers. An additional image showing staining performed with FFBP 30 min post coagulation is shown in P). Further controls show representative images acquired from a dish coated with type I (rat rail) collagen in the presence (Q) and absence (R) of RBCs. All images were obtained using a 63× oil immersion objective with a numerical aperture of 1.42. Scale bar = 16 μm. All experiments were performed at least three times, and representative samples are shown.

2 – Fibrin binding under static conditions with variable concentrations of fibrinogen (FGTW, LFB Biomedicines, Les Ulis, France), red blood cells (RBCs) and platelets (Figure 2).

**Figure 3:**
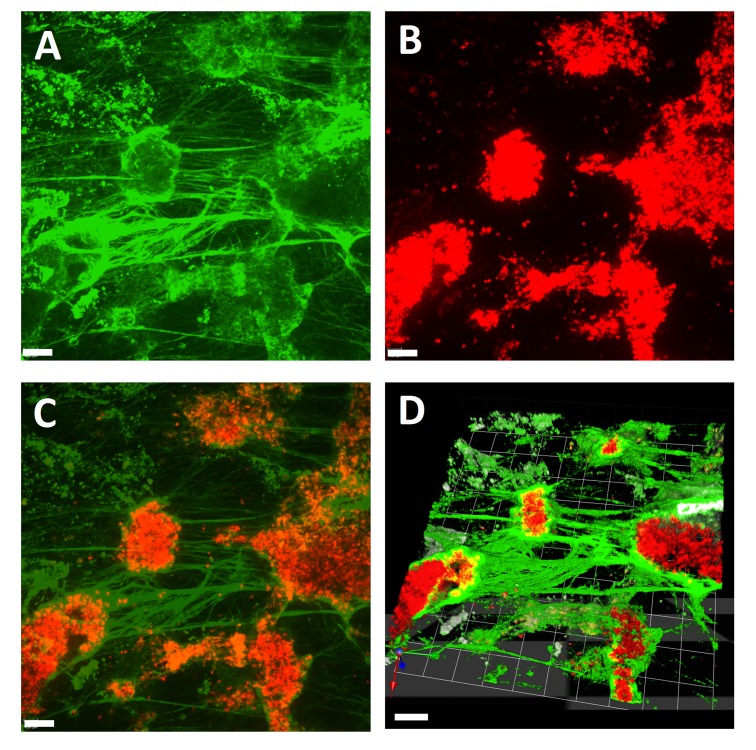
Visualization of clot formation induced by star-tem and ex-tem in the Cellix microfluidic biochip system. A) Fibrin stained with FFBP, B) RBCs stained with wheat germ agglutinin and C) a merged image of A and B. D) A 3-dimensional representation of the same clot made by combining a Z-stack of images A and B. Scale bar = 10 μm. The round objects stained by wheat germ agglutinin are platelet aggregates. All experiments were performed at least three times, and representative samples are shown.

3 – Fibrin binding in a simulated flow condition (Figure 3).

**Figure 4:**
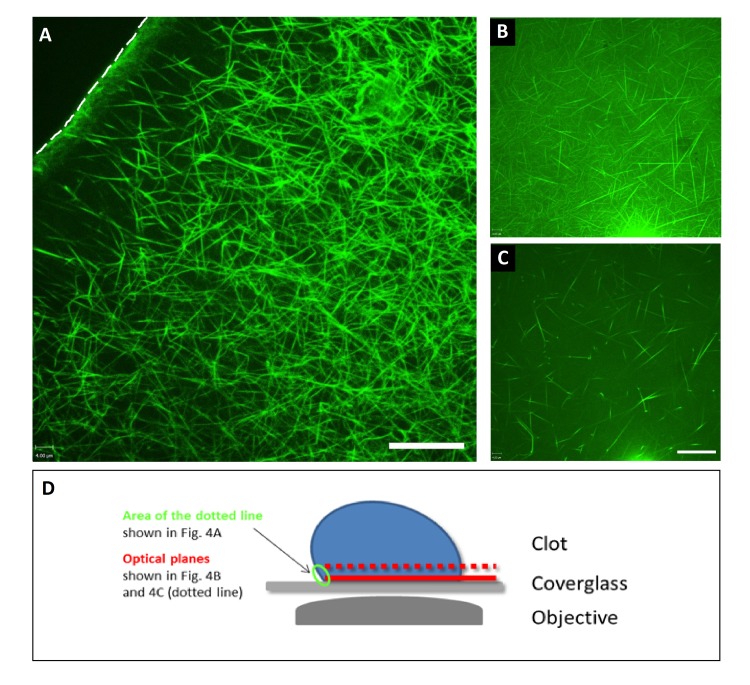
A) Fibrin formation in response to air (top left corner of the image) as visualized via FFBP under static conditions in an 8-well chamber. Clot formation was induced by addition of star-tem and ex-tem. Shown is the border of the clot that faces the air (dotted line). Note the dense architecture of the fibrin network at this border compared to the architecture of the network at the center of the clot. Notably, staining of the fibrin network was performed after its formation. B) Fibrin architecture at the bottom of the 8-well slide and C) 1 µm away towards the center of the clot. D) Shows a diagrammatic representation of how images A-C were obtained. Note the different architecture which resembles the one shown before at the contact site towards the air. Scale bar = 16 μm. All experiments were performed at least three times, and representative samples are shown.

4 – Post-staining; fibrin binding of an existing fibrin net-work created under static conditions (Figure 4).

### 2.3 Blood/platelet-rich plasma/plasma samples

Blood was drawn by clean venipuncture and collected into vacutainer tubes containing

1/10 volume of 3.12% trisodium citrate as an anticoagulant (Sarstedt, Nümbrecht, Germany).

Platelet-rich plasma (PRP) was obtained by centrifugation of whole blood at 200 g for 20 min. Erythrocytes were collected from the bottom fraction and adjusted to the different numbers with plasma obtained after the second centrifugation to prepare PRP. To prepare plasma samples, PRP was centrifuged at 14000 g for 5 min. Platelet number was determined using a Coulter hematology analyzer. Volumes were equalized for all samples with autologous plasma (i.e., same volumes for all samples).

### 2.4 Live confocal assessment of FFBP fibrin-binding

Visualization of FFBP fibrin-binding under static conditions was performed in Lab-Tek 8-well chambered #1.0 borosilicate cover glass slides (Nunc, Rochester, NY, USA). For this purpose, 200 µl of either citrated plasma only or with added erythrocytes/platelets were pipetted into each well. Coagulation was induced via addition of 5 µl star-tem (0.2 mol/l CaCl_2_ in HEPES buffer, pH = 7.4) and 5 µl ex-tem (recombinant tissue factor; Tem Innovations GmbH, Basel, Switzerland).

FFBP fibrin-binding under dynamic conditions was per-formed and visualized in a flow chamber model (Vena8 Endothelial biochips, Cellix; Dublin, Ireland). As in the static model, coagulation was induced by addition of star-tem and ex-tem. The applied shear stress was 60 dyne/cm^2^. Clots were formed in the presence of FFBP. Star-tem and ex-tem were added to the chamber which is placed before the vessel.

Real-time live confocal microscopy was performed with a spinning disk confocal system (UltraVIEW VoX; Perkin Elmer, Waltham, MA, USA) connected to a Zeiss AxioObserver Z1 microscope (Zeiss, Oberkochen, Germany). Images and Z-stacks were acquired using Volocity software (Perkin Elmer) using a 63× oil immersion objective with a numerical aperture of 1.42.

Sensitivity and specificity of FFBP for visualizing clot formation were compared with pre-labeled fibrinogen Alexa Fluor 647 (Thermo Fisher Scientific, Waltham, MA, USA) under static conditions in the 8-well chambered cover glasses as described above. Red blood cells (RBCs) and platelets were visualized using wheat germ agglutinin-Alexa Fluor 555 (WGA; Thermo Fisher Scientific). Each well received 200 μl plasma +/- red blood cells/platelets, 5 μl ex-tem (recombinant tissue factor and phospholipids, heparin inhibitor, preservatives and buffer, 5 μl star-tem (0.2 mol/l CaCl_2_ in HEPES buffer) (ROTEM, Basel, Switzerland), FFBP solution (final concentration 0.0045 μg/μl) and fluorescently pre-labeled fibrinogen (final concentration 0.015 μg/μl). The effects of various concentrations of fibrinogen (FGTW, LFB Biomedicines, Les Ulis, France), RBCs and platelets on clot fibrin networks were imaged by real-time live confocal microscopy as described above.

### 2.5 Data analysis and presentation

All experiments were performed with at least three replicates. No statistical analyses were performed, but for each experiment at least five independent areas were imaged; representative images for each experiment are shown.

### 2.6 Ethics statement

This study was approved by the human subjects review board of the Medical University of Innsbruck, Austria (Ref. No. UN4984_LEK), and by the national competent authority (Bundesamt für Sicherheit im Gesundheitswesen, Vienna, Austria). Written informed consent was obtained from all study participants. The study was performed in compliance with the Declaration of Helsinki and followed the Good Clinical Practice guidelines as defined by the International Conference on Harmonization (ICH-GCP).

## 3 Results and discussion

### 3.1 FFBP peptide development

The structure of the FFBP is based on previous studies de-scribing fibrin-binding peptides [9-12], and the specific fibrin-binding motif of FFBP was described by Overoye-Chan and coworkers [10], who modified a previous structure by addition of three unnatural amino acids. This modification improved the fibrin-binding affinity, with the resulting motif binding two equivalent sites on human-, mouse-, rat-, rabbit-, pig- and dog-derived fibrin with high affinity (*K*_d_ = 1.7 ± 0.5 μM) and specificity (100- and 1000-fold higher binding to fibrin than fibrinogen and albumin, respectively) [10]. The excellent binding parameters prompted us to use this specific peptide, fusing it with FITC at the N-terminus to create the fluorescent FFPB. Following synthesis of the fluorescently linked peptide, we proceeded to assess whether fibrin-binding characteristics were retained following fusion with FITC.

### 3.2 In vitro real-time live confocal imaging of fibrin with FFBP

*Specificity of fibrin staining with FFBP –* Initial imaging in 8-well chambered cover slides showed excellent overlap between FFBP and Alexa Fluor 647-labeled fibrinogen in citrated human plasma (Figure 1). In this figure, we added RBCs and platelets to the plasma sample in order to show the specificity of FFBP for fibrin. The cellular components are visualized via bright field microscopy (Figure 1C). The overlay of both fluorescent fibrin stains (FFBP; Figure 1A) and (fluorescently labeled fibrinogen; Figure 1B) with the bright field image is shown in Figure 1D. These results demonstrate that the fibrin-binding characteristics of the original peptide were not affected by fusion with FITC. No cross reactivity was ob-served. The real-time formation of fibrin using FFBP is shown in a video in the supplementary materials (S1 and S2).

*Visualization of fibrin formation via FFBP under static conditions –* After demonstrating the specificity of FFBP, we proceeded to test whether FFBP enabled fibrin to be visualized under different conditions. For this purpose we used human plasma and spiked it with varying concentrations of fibrinogen (1-50 µg/ml), RBCs (5.5×10^4^-2.7×10^6^/well) and platelets (1.5×10^4^-7×10^5^/well) (Figure 2). As can be seen in Figure 2A-D, increasing the fibrinogen concentration results in a higher fibrin network density. The contrary is the case when RBCs are added (Figure 2E-H), and may be due to the fact the higher volume of RBCs prevents the formation for platelets, which can only be formed in plasma surrounding the RBCs. Addition of platelets results in higher numbers of interconnections between the fibrin fibers (Figure 2I-L). Controls showing the images acquired when adding the labeling 15 minutes after coagulation induction are also presented (Figure 2M-O). An additional image demonstrates FFBP-labeling 30 minutes post coagulation (Figure 2P). Notably, FFBP provides good staining even when added after clot formation although there appears to be a slight increase in background fluorescence. Additional controls show representative images acquired from a dish coated with type I (rat rail) collagen in the presence (Figure 2Q) and absence (Figure 2R) of RBCs.

*Fibrin visualization with FFBP in simulated flow conditions –* After showing the versatility of FFBP and real-time live confocal imaging under static conditions, we proceeded to characterize the binding of FFBP to fibrin under more naturalistic conditions and obtained excellent visualization of clot formation induced by star-tem/ex-tem under conditions of simulated flow, and were able to produce high-quality 3-dimensional representations of the clot by combining image Z-stacks (Figure 3). The background staining seen in some parts of this figure may be attributable to microparticle-triggered fibrin formation.

*FFBP post-staining of an existing fibrin network at the surface/air borderline of a clot –* Next, we investigated fibrin formation in response to air, which might influence clot formation under conditions of traumatic bleeding (Figure 4A; see also the diagrammatic explanation of the experiment in Figure 4D). Also, here we were able to obtain clear visualization of a fibrin mesh using FFBP, which in this case was added after clot formation, indicating the peptide successfully binds fibrin under a wide range of conditions. Note the dense architecture of the fibrin network at this border (Figure 4B) compared to the architecture of the network towards the center of the clot (Figure 4C). Importantly, as the FFBP was applied after expo-sure to air and clot formation, this demonstrates the ability of FFBP to bind fibrin after it has formed and therefore presents an alternative method for conveniently post-staining fibrin in a wide range of models/situations. The heterogeneous densities of the fibrin network is also shown in a video scanning the Z-axis (of the same area shown in Figure 4) which is available as a supplementary video (S3).

### 3.3 FFBP highlights fibrinogen as a key factor in coagulation

Fibrinogen, also known as Factor I, is present in healthy individuals at a concentration of 263 mg/dl, making it one of the most abundant plasma proteins [13]; quantitatively it is amongst the most prominent proteins involved in coagulation [14]. Furthermore, abnormalities in fibrin levels are associated with a range of symptoms including heart disease and inflammation [15-17]. Our results highlight the importance of fibrinogen and fibrin in the clot formation process. However, further work is needed to determine how fibrin interacts with its anatomical and biochemical environment under specific *in vivo* conditions. For this, high-quality models and tools need to be further progressed [5]. The development and characterization of FFBP should be viewed as an essential step in this process.

### 3.4 Advantages of FFBP as a tool for studying fibrin(ogen) interactions

Until now, microscopic investigation of fibrin formation has generally employed pre-labeled fibrinogen to visualize fibrin structures [6,18]. The FFBP peptide used here has been described previously in combination with a different fluorophore. In that case it demonstrated significant binding to ferric chloride-induced thrombi [9]. We extend these findings by combining the same fibrin-binding peptide with a different fluorophore and using it in the context of real-time live confocal imaging. The peptide concentrations used in our experiments were similar to those described by Hara et al. [9]. Our results show that fibrin visualized with FFBP overlaps with Alexa Fluor 647-labeled fibrinogen. This indicates that FFBP is capable of binding fibrin, both in an *in vitro* cover slide model as well as in simulated flow under conditions similar to those observed in traumatic bleeding. However, the exact binding affinities for fibrin/fibrinogen remain to be determined. The use of FFBP instead of pre-labeled fibrinogen for labeling fibrin is advantageous for several reasons. The primary ad-vantage is that it allows for the labeling of fibrin networks after they have been formed. Although other methods exist for post-staining fibrin networks using antibodies, these methods generally require fixation of the sample prior to the use of primary and secondary antibodies [19], making the process more complex and less suitable for, in particular, *in vivo* use. Second, it avoids any threats to validity posed by the potential interference of fluorescent fibrinogen tags with the delicate process of fibrin polymerization.

A possible limitation of FFBP is that its post-staining abilities may be limited by reduced clot permeability due to the presence of RBCs or other cells around the clot. Such a potential issue may be addressed by using other stains, such as fluorescently labeled WGA in order to show how well or not the clot can be permeated with fluorescent stains.

## 4 Future directions

We believe that the properties and characteristics of FFBP described in this report make it particularly valuable for use in combination with intravital confocal microscopy. Intravital microscopy is a promising tool for biomedical research in general and, in particular, for studying thrombus formation, for instance in the context of traumatic bleeding. Although fibrinogen is important during traumatic injury, its interaction with other hemostatic processes, such as injury site vasoconstriction and the formation of extraluminal soft clots, has mostly been overlooked and is not well understood [20-22]. Recently, a new and promising intravital bleeding model was described [23], and future work should aim to employ the FFBP in this model. We recently demonstrated the utility of the confocal microscopy approach for imaging biopsies to assess organ function [24,25], and are currently working to develop an *in vivo* model of cut vessel flow that allows for investigation of hemostasis following traumatic bleeding that employs FFBP-facilitated intravital confocal visualization of fibrin.

## 5 Conclusion

The FITC-linked Fibrin-Binding Peptide (FFBP) is a valuable tool for confocal microscopic assessment of fibrin(ogen) that has clear and specific advantages over the use of pre-labeled fibrinogen. Work to assess the value of this tool in an intravital model of traumatic bleeding is currently ongoing.
